# Effects of different types of leisure activities on working memory across the adult lifespan

**DOI:** 10.1007/s00426-024-01998-x

**Published:** 2024-07-06

**Authors:** Selene Cansino, Frine Torres-Trejo, Cinthya Estrada-Manilla, Silvia Ruiz-Velasco

**Affiliations:** 1 Laboratory of NeuroCognition, Faculty of Psychology, National Autonomous University of Mexico, Avenida Universidad 3004, Building D, Room 212, 04510, Mexico City, México; 2 Applied Mathematics and Systems Research Institute, National Autonomous University of Mexico, Mexico City, Mexico

## Abstract

The aim of the present study was to contrast the potential influence of five independent types of leisure activities (physical, mental, social, cultural, and passive) on working memory in a lifespan sample and in specific stages of adulthood (young, middle-aged, and older adults). A sample of 1652 healthy adults between 21 and 80 years of age participated in the study. Leisure activities were assessed through a lifestyle questionnaire created for the study. Working memory was measured in the verbal and spatial domains using a computerized n-back task that allowed us to reliably measure discrimination and reaction times. Across adulthood, mental (computer use and hobbies) and social leisure activities predicted greater verbal and spatial working memory discrimination; mental (reading) and social activities predicted faster verbal working memory; and mental (computer use) and physical activities predicted faster spatial working memory. In young adults, mental (computer use) and social activities were associated with greater verbal and spatial working memory performance. In middle-aged adults, physical and mental activities (computer use) were associated with greater working memory performance. In older adults, physical, mental (hobbies), and social activities were associated with greater working memory performance. Leisure activities can enhance working memory discrimination and speed independent of individuals’ age.

**Supplementary Information:**

The online version contains supplementary material available at 10.1007/s00426-024-01998-x.

## Introduction

Leisure activities refer to all activities that individuals engage in during their free time for pleasure. They are clearly distinguished from activities performed for work or other responsibilities. Studies have revealed that the way we spend our free time influences our cognitive functioning (for a meta-analysis, see Yang et al., 2022). However, little is known about which recreation activities have a greater impact on cognition and, specifically, which type of cognitive ability benefits from which types of pleasure activities. This information is lacking because most studies have exclusively examined the effects of one free-time activity on cognition (e.g., Litwing et al., 2017; Willey et al., 2016) or because most studies that have assessed several spare activities used composite measures that combined several types of activities (e.g., Sala et al., 2019; Zhu et al., 2022). Therefore, research in this field requires a more specific methodological approach to increase the understanding of leisure activities and their impact on cognition.

One of the most common leisure activities investigated is physical activity. A systematic review (Engeroff et al., 2018) reported a total of 23 cross-sectional and longitudinal studies that examined the engagement of individuals over the age of 18 in physical activity and its effects on a specific cognitive domain in individuals over the age of 60. The results revealed that moderate and vigorous physical activity was associated with better global cognitive function and better executive function. Similarly, a longitudinal study (Willey et al., 2016) of 876 older adults found that after five years, processing speed and episodic memory declined more in individuals who performed light-intensity physical activity or none at all than in those who performed moderate- to high-intensity physical activity. Another study (Phansikar & Mullen, 2020) that compared 579 older adults who performed physical activity and 1790 sedentary persons revealed that the former group exhibited superior processing speed, verbal fluency, and delayed recall than did the latter group.

On the other hand, mental leisure activity refers to activities that require a high level of cognitive effort. Some activities that are considered part of this category are reading, using a computer, or engaging in hobbies. A previous study (Ferreira et al., 2015) conducted through an online platform examined 65,431 participants between 18 and 90 years old. This study revealed that the frequency with which participants engaged in crosswords, Sudoku, and puzzles was associated with better spatial working memory and episodic memory scores. These scores were also higher for individuals under the age of 65 who engaged in computer games more often. Similarly, a longitudinal study (Litwin et al., 2017) of 16,572 participants older than 65 from 20 European countries assessed the frequency with which participants performed word or number crossword puzzles or Sudoku in the previous 12 months. Individuals who performed these mental activities more often had better memory, numeracy, and fluency two years later.

The effects of social leisure activities on cognition were examined in a sample of 237,644 individuals over 50 years old from 20 European countries (Christelis & Dobrescu, 2020). Social activity in this study was classified as no activity, one activity, or two or more activities. The activities considered were engaging in voluntary work, attending courses, playing sports, or attending social clubs. This study revealed that social engagement had positive effects on numeracy, fluency, and immediate and delayed recall. A systematic review (Kelly et al., 2017) of 22 studies of healthy adults aged 50 years or older concluded that social activities improved speed, working memory, and executive functions but not attention or episodic memory.

One leisure activity that has been overlooked is attending cultural events, with the exception of one study (Fancourt & Steptoe, 2018) that analyzed data from 3445 adults over the age of 52. This study found that visiting museums, galleries, or exhibitions and attending the theater, concert, or opera benefited verbal memory 10 years later, as measured through composite scores of immediate and delayed memory. In another study (Konlaan et al., 2000) not related to cognition, 10,609 Swedish individuals aged 25–74 years were interviewed. Individuals who attended cultural events at least occasionally were found to have a lower mortality risk 14 years later than those who did not attend cultural events. Some leisure activities, such as watching TV and listening to the radio, are characterized as passive because they do not require mental effort, although some authors (e.g., Hassing et al., 2020) classify them as mental leisure activities. The controversy about whether these activities should be considered passive or mental has been intensified by the fact that results of the effects of these activities on cognition are highly discrepant. A study (Mao et al., 2019) of 10,741 healthy individuals over 80 years old revealed that watching TV almost every day and listening to the radio were associated with a lower risk of cognitive impairment 3.4 years later. In contrast, a study (Raichlen et al., 2022) of 146,651 healthy individuals with a mean age of 65 years revealed that the time spent watching TV was associated with an increased risk of all-cause dementia.

Most studies (e.g., Gow et al., 2014; Sala et al., 2019) that have analyzed several leisure activities used composite measures; thus, the effects of each activity independently on cognition are unclear. Two studies (Sanz Simon et al., 2023; Wang et al., 2012) compared the effects of physical, mental, and social leisure activity, but the results were not consistent. One of these studies (Sanz Simon et al., 2023) reported that only mental activity predicted greater memory preservation five years later, whereas the other (Wang et al., 2012) reported that only physical activity predicted greater episodic memory preservation 2.4 years later. The discrepancy between these results could be attributed to methodological differences; for example, Sanz Simons et al. (2023) examined a memory score that included different types of memory in a sample of 208 individuals between 21 and 80 years old, whereas Wang et al. (2012) examined episodic memory in 1463 individuals 65 years of age or older. Nevertheless, which type of leisure activity has a stronger effect on specific cognitive domains remains an open question.

According to the literature reviewed, mental (Ferreira et al., 2015) and social (Kelly et al., 2017) leisure activities are positively associated with working memory performance. However, the direct contrast between the effects of different leisure activities on working memory has not been previously evaluated. In the present study, we focused on working memory because it is the memory process that we employ constantly during our waking lives and is essential for dealing with everyday demands, such as following a conversation, planning actions, or solving all kinds of problems. Working memory refers to the ability to maintain and transform information that briefly remains active in the conscious mind (Baddeley & Hitch, 1974; Cowan, 1999). Moreover, working memory shows a critical decline across adulthood (Cansino et al., 2013; Park et al., 2002). Thus, it is important to identify leisure activities that may prevent this decline. We examined working memory in the verbal and spatial domains with an n-back task (D’Esposito et al., 1998; Kirchner, 1958) at a 2-back level of difficulty that requires the main processes that characterize working memory, which are storage, binding, retrieval, updating, monitoring, and interference control. The operation span task was not used because this task does not allow us to examine working memory performance in different domains, such as the n-back task, which allowed us to examine verbal and spatial working memory. This distinction was important in the present study because we were interested in determining whether specific leisure activities might be associated with working memory performance in each of these specific domains. Moreover, there are substantial differences between both tasks, as revealed by a meta-analysis (Redick & Lindsey, 2013), and we chose the 2-back task because it clearly measures the main processes that characterize working memory. The n-back tasks employed in the present study follow the classically validated characteristics and parameters employed by numerous studies, as described in a meta-analysis that included 24 studies (Owen et al., 2005). We used a computerized task that allowed us to reliably measure working memory accuracy and speed. The effect of leisure activities on working-memory speed has not been previously investigated.

As described, most related studies have been performed with older adults, except for one study that examined middle-aged adults through composite leisure activity scores involving low and high cognitive effort and individual and social activities. Only high cognitive effort and social leisure activities were positively associated with cognition. One study (Sanz Simon et al., 2023) examined a lifespan sample, but the effect of leisure activity was analyzed together in the whole sample rather than at specific life stages. Therefore, the effects of leisure activities on young adults have not previously been examined independently of other age groups. The aim of the present study was to assess the effects of physical, mental, social, cultural, and passive leisure activities on the accuracy and speed of verbal and spatial information processing within working memory across adulthood and in the main stages of life (i.e., young, middle-aged, and older adults). The present study is the first to evaluate the effects of five different leisure activities on working memory in the same lifespan sample, which allows direct comparisons of their individual effects on each life stage under the same conditions.

We hypothesized that physical leisure activity would be associated with better working memory performance because a meta-analysis of randomized controlled trials revealed that physical activity in older adults improved working memory (Zhidong et al., 2021). Additionally, based on previous findings, we expected that mental (Ferreira et al., 2015) and social (Kelly et al., 2017) leisure activity would be associated with better working memory performance. Although the influence of cultural leisure activity on working memory has not been examined previously, we expected positive effects of this leisure activity on working memory, as observed for general memory scores (Fancourt & Steptoe, 2018). Due to the contradictory findings observed for the effects of passive leisure activity on general cognition (Mao et al., 2019; Raichlen et al., 2022), we did not expect an influence of this leisure activity on working memory. Because the effects of specific leisure activities on working memory have not been previously examined in young and middle-aged adults, we had no a priori predictions for these age groups. Likewise, we expected that physical, mental, social, and cultural leisure activities would be associated with faster working memory processing, even though this variable has not previously been assessed.

## Methods

### Participants

An adult life span sample of 1652 healthy adults aged between 21 and 80 years participated in the present study. We recruited 1657 participants; however, five individuals were excluded because their data were lost due to technical difficulties. The sample size was not calculated a priori and was determined based on financial resource constraints. Participants were invited to the study through appeals to community groups, advertisements, flyers and word of mouth. To be included in the study, participants had to have at least eight years of education, normal or corrected-to-normal vision, a score ≤ 20 on the Beck Depression Inventory (BDI) (Beck, 1987), a score ≥ 24 on the Mini Mental State Examination (MMSE) (Folstein et al., 1975), and a score ≥ 26 on the vocabulary subtest of the Wechsler Adult Intelligence Scale-Revised (WAIS-R) (Wechsler, 1981). These scores were used to guarantee that the participants were not suffering from depression, dementia, or intellectual difficulties. Participants with these affections were excluded because the comparison between age groups would not be reliable and because we planned to answer our experimental questions in mentally healthy individuals. Likewise, all participants were free of neurological and psychiatric disorders. None of them had taken medication that affected the central nervous system in the last six months, none were addicted to drugs or alcohol, and none had suffered head trauma. The entire sample was divided into three groups according to the participants’ ages: young (21–40 years), middle-aged (41–60 years) and older (61–80 years) adults. The characteristics of the participants in each of these groups and the entire sample are displayed in Table 1. The study was approved by the bioethics committee of the School of Medicine at the National Autonomous University of Mexico. The experiments were performed in accordance with the Declaration of Helsinki. All participants provided informed consent and received a monetary reward for their participation.

**Table 1:** Participants’ characteristics, scores on neuropsychological tests, and performance in the verbal and spatial working memory tasks for the whole sample and by adulthood stage

	All		Young		Middle-aged		Older	
	Mean/number	SD	Mean/number	SD	Mean/number	SD	Mean/Number	SD
Participants	1652		527		537		588	
Sex (female/male)	831/821		263/264		271/266		297/291	
Age (years)	51.02	17.71	29.56	6.26	50.82	5.32	70.45	5.44
Education (years)	14.52	4.23	15.93	2.79	14.66	4.53	13.14	4.58
Vocabulary (WAIS-R)	12.71	1.71	12.76	1.59	12.63	1.70	12.74	1.82
MMSE	28.54	1.38	29.01	1.13	28.57	1.32	28.09	1.49
BDI	6.61	5.08	6.09	5.03	6.39	4.96	7.29	5.15
Verbal WM (d’)	2.14	0.91	2.68	0.78	2.08	0.87	1.71	0.79
Spatial WM (d’)	1.68	0.93	2.23	0.85	1.64	0.85	1.23	0.81
Verbal WM RT (ms)	1137	239	1047	227	1151	223	1205	238
Spatial WM RT (ms)	1240	260	1155	252	1263	244	1294	263

*WAIS-R* Wechsler adult intelligence scale-revised, *MMSE* mini mental state examination, *BDI* beck depression inventory, *WM* working memory, RT reaction time

### Measures

The BDI is a 21-item self-report questionnaire that evaluates the severity of depressive symptoms by asking questions about depressive feelings experienced in the past week. Each item has four response options that differ in intensity. The MMSE is used to assess the presence of cognitive impairment or dementia. It contains 11 simple questions that are administered to the participant to examine orientation, concentration, attention, calculation, memory, and language. The Vocabulary subtest of the WAIS-R indicates the intellectual integrity of an individual because its scores are highly correlated (r = 0.81) with the full-scale intellectual quotient (IQ) (Cullum, 1998). The subtest is composed of 40 words that participants are asked to define, but after five consecutive words with a zero score, the application is ended.

We created a questionnaire to examine several lifestyle variables. The questionnaire assessed the frequency and duration of the participants’ involvement in physical activity (aerobic and anaerobic exercise), watching television, listening to the radio, using the computer, reading and hobbies. Additionally, the participants reported their frequency of attending cultural events (film screenings, theater plays, exhibitions, concerts, conferences or courses) and social events (parties or reunions). Frequency was divided into 10 categories (never, once a year, three times per year, six times per year, once per month, two or three times per month, one or two times per week, three or four times per week, almost every day and daily). The participants also reported the type of exercise they performed most frequently and the genres of television, radio and literature they most often chose. Similarly, they were asked to report the kind of activity they most frequently engaged in on the computer and the types of hobbies in which they engaged. The leisure activities selected herein are representative of the main free-time activity categories (physical, mental, social, cultural, and passive) outlined in the literature. The division of frequency into 10 categories was performed ad hoc with the purpose of measuring all possible frequency patterns, from never to daily. The questionnaire was administered as a semistructured interview in which the 10 frequency categories were displayed on a sheet of paper placed in front of the participant. Participants were asked how frequently they performed each activity by selecting one of the 10 frequency categories. Then, participants were asked to indicate the time they spent engaged in the specific activity, followed by the question of which specific type or genre they most often chose for that specific activity.

### Stimuli

We used 12 uppercase letters (B, F, G, K, L, N, P, Q, R, S, T and X) to examine verbal working memory in the n-back task. The letters were selected randomly from the entire alphabet with the only constraint to be a consonant. We selected 12 letters to maintain the same number of stimuli as in the spatial n-back task, in which 12 possible positions were used. The letters were presented at the center of the screen with a vertical visual angle of 1.5° and a horizontal visual angle of approximately 1°. The letters were presented in a dark gray color on a white screen to maintain low contrast. To assess spatial working memory with the n-back task, we used a dark gray circle with a diameter visual angle of 1.5°. The circle was displayed in one of 12 possible positions around the center of a white screen, as in an imaginary clock. The distance between the circle and the center of the screen was 4°. A black cross (vertical and horizontal visual angles of 0.5°) was continuously displayed at the center of the screen. The letters and positions for the verbal and spatial tasks were selected randomly and with the same probability.

### Procedure

Each participant attended two sessions that were each approximately two hours long. Prior to being invited to attend the first session, potential participants were asked prescreening questions to determine whether they fulfilled the inclusion criteria. The first session was conducted in a silent room in which only the participant and the experimenter were present. In this session, the participants were interviewed to further determine whether they satisfied the inclusion criteria. Then, the participants completed the WAIS-R Vocabulary subtest, the MMSE and the BDI, and their vision was tested. Participants who were suitable for the study were asked to provide informed consent. Subsequently, the participants were further interviewed about their lifestyle and leisure activities. The second session took place in a sound-dampened chamber approximately 1 week after the first session. In this session, the participants performed the working memory tasks and a source memory task. We employed a source memory paradigm, introduced by Cansino et al. (2002), that measures recollection processes in episodic memory. However, the data obtained for this task was not included because it was beyond the scope of this paper. The participants performed these tasks while seated in a high-back armchair located 100 cm from the monitor screen (1. The participants responded by utilizing two keys on a response panel located on a left or right platform placed on the arm of the chair at a comfortable distance according to the participants’ handedness. The response panels were constructed for the experiment. The participants performed the verbal and spatial n-back tasks in counterbalanced order. Within each domain, participants performed the task at two levels of difficulty (one-back and two-back), also in a counterbalanced order. Data from the low-difficulty tasks (one-back tasks) are not shown because this task does not demand all the processes that characterize working memory (Oberauer, 2005), and some participants’ performance showed ceiling effects. Prior to performing each of the four n-back tasks, the participants completed brief versions of each task as training. The experiments were run on a computer with an Intel Core i7 processor and Windows 7 operating system. The tasks were conducted with the software E-Prime v1.0 from Psychological Software Tools (Pittsburgh, PA, USA).

### Verbal working memory paradigm

**Fig. 1:**
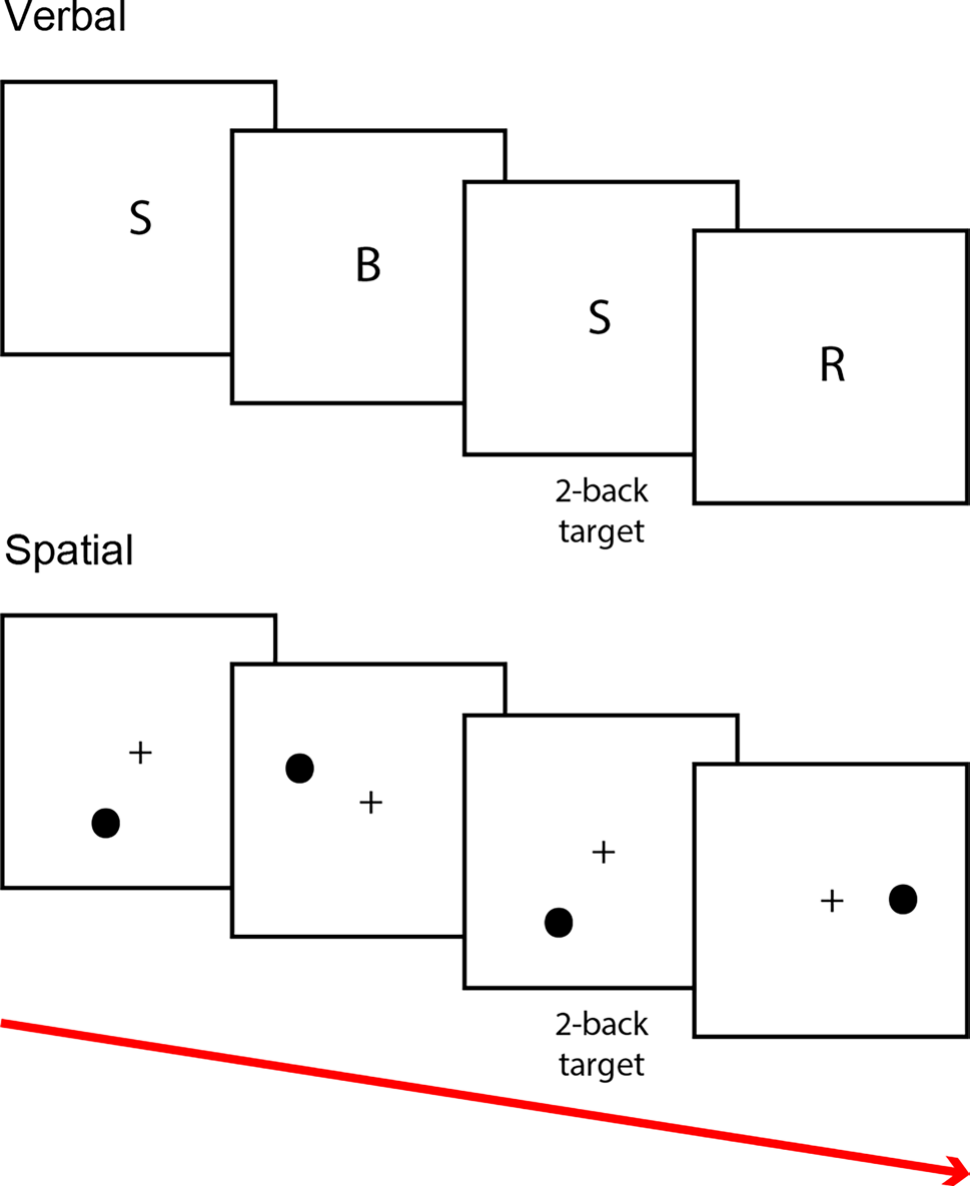
Events during the verbal and spatial working memory tasks

Each trial started with the presentation of the letter for 300 ms at the center of the screen, followed by a blank period of 2700 ms (Fig. 1). Then, the next stimulus was displayed. The participants were allowed to provide their response during the 3000-ms period following the onset of the stimulus. The participants were asked to indicate whether the current letter was equal or not equal to the one displayed two trials prior. The participants performed 72 trials, 33% of which were target trials (letters equal to those of the current trial).

### Spatial working memory paradigm

At the beginning of each trial, a circle was displayed for 300 ms in one of the 12 positions surrounding a cross that was continuously presented at the center of the screen (5° of vertical and horizontal visual angle). Then, after 2700 ms, the next stimulus was displayed. The task consisted of indicating whether the current circle was presented in the same position as the one displayed two trials earlier. The participants were allowed to respond during a period of 3000 ms following the onset of the stimulus. Each participant performed 72 trials, 33% of which were target trials (positions equal to those of the current trial).

### Data analysis

All variables were analyzed with descriptive statistics and bivariate correlations. The data from the first two trials of the 2-back tasks were excluded from the analysis because a target did not occur in these trials. The ability of each participant to separate a signal from noise was determined for each task using d-prime values. The d-prime values were selected for analyses because they provide an accurate estimation of the participants’ level of discrimination independent of the criterion for completing the task (Macmillan & Creelman, 1990). Reaction times for correct responses in each working memory task were measured from the onset of stimulus presentation until the participant’s response. Working memory discrimination and speed differences across age groups were assessed through one-way analyses of variance (ANOVAs). Total time (hours/week) was estimated as the product of frequency and duration of physical activity, use of the computer, reading, hobbies, watching television, and listening to the radio, independent of the specific type or genre. Multiple linear regression models were constructed to examine the association between leisure activities and memory performance. These models were constructed separately for discrimination levels (d’ values) and reaction times in the verbal and spatial working memory tasks. Multiple regression models were first computed on data from the whole sample and then for each age group (young, middle-aged and older adults). All leisure activities (physical activity, using the computer, reading, hobbies, social activities, cultural activities, watching television, and listening to the radio) were entered simultaneously in the model and were treated as predictors, while discrimination and reaction times in the verbal and spatial tasks were the outcomes. Adjustments were made for age, years of education, vocabulary, MMSE score and BDI score.

One-way ANOVA was conducted for each leisure activity across the three age groups to estimate whether leisure activities changed across the lifespan. Significant differences among age groups were further analyzed by Tukey’s honest significant difference (HSD) test. All analyses were conducted using Stata version 16 (Texas, USA).

## Results

The results of the descriptive statistics for all variables are displayed in Supplemental Material Table S1. The participants’ performance in the verbal and spatial working memory tasks is shown in Table 1, which includes the participants’ discrimination levels and reaction times obtained in the entire sample and in each age group. Differences in verbal [*F*(2, 1649) = 198.22, *p* < 0.001, μ^2^ = 0.19] and spatial [*F*(2, 1649) = 195.67, *p* < 0.001, μ^2^ = 0.19] working memory discrimination and in verbal [*F*(2, 1649) = 67.25, *p* < 0.001, μ^2^ = 0.08] and spatial [*F*(2, 1649) = 45.10, p < 0.001, μ^2^ = 0.05] working memory reaction times were observed across all groups, except between middle-aged and older adults in spatial working memory speed, according to Tukey HSD tests. The correlation matrix for the models containing the entire sample is depicted in Supplemental Material Table S2. The variance inflation factors (VIFs) for each leisure activity were as follows: physical activity, VIF = 1.01; using the computer, VIF = 1.11; reading, VIF = 1.11; hobbies, VIF = 1.02; social activities, VIF = 1.07; cultural activities, VIF = 1.15; watching television, VIF = 1.05; and listening to the radio, VIF = 1.01.

**Fig. 2:**
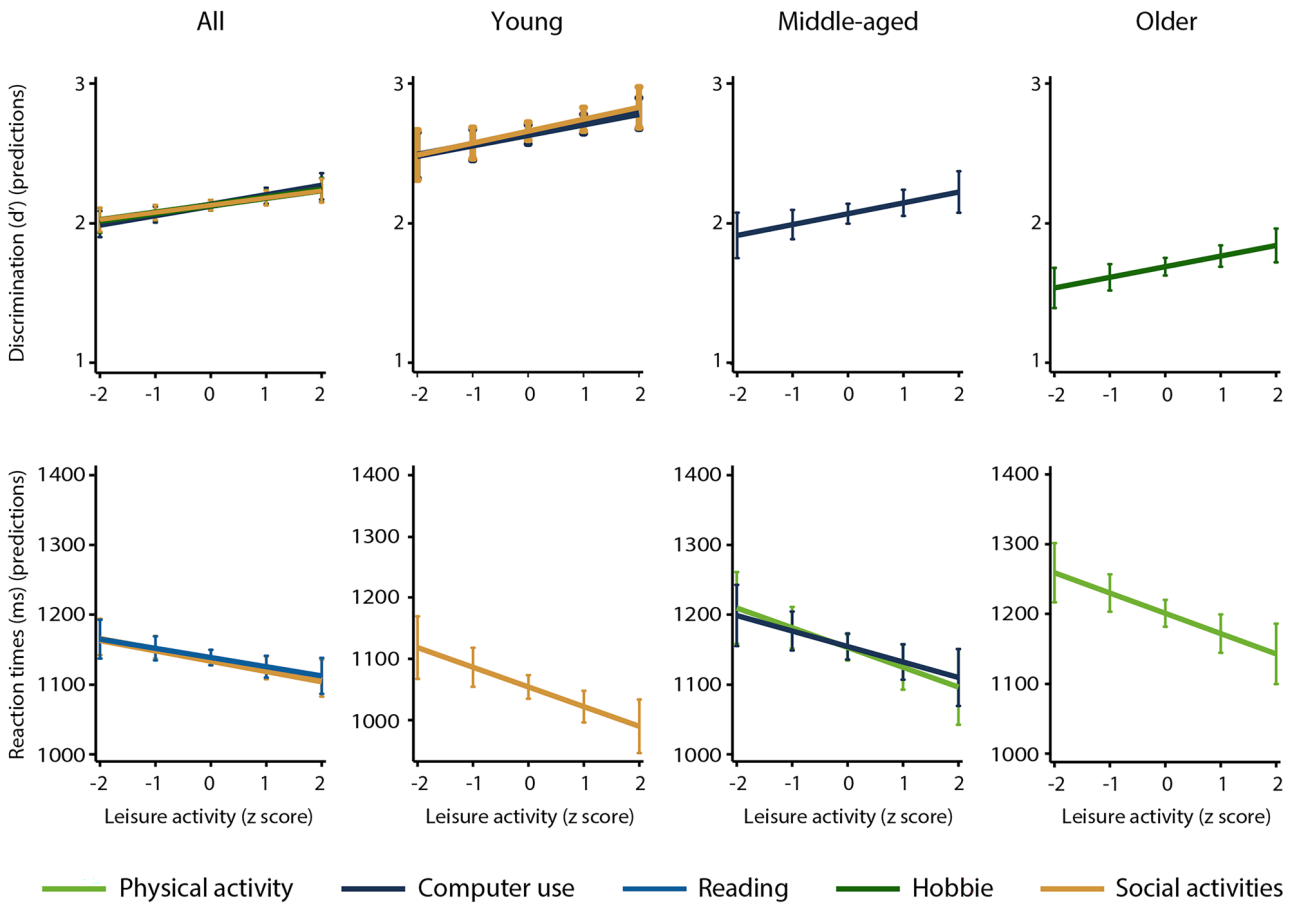
Regression plots for leisure activities that significantly predicted discrimination levels and reaction times for verbal working memory in the entire sample and in each age group. Leisure activities are depicted in z scores to allow for comparison. Error bars represent the 95% confidence intervals of the mean

**Fig. 3:**
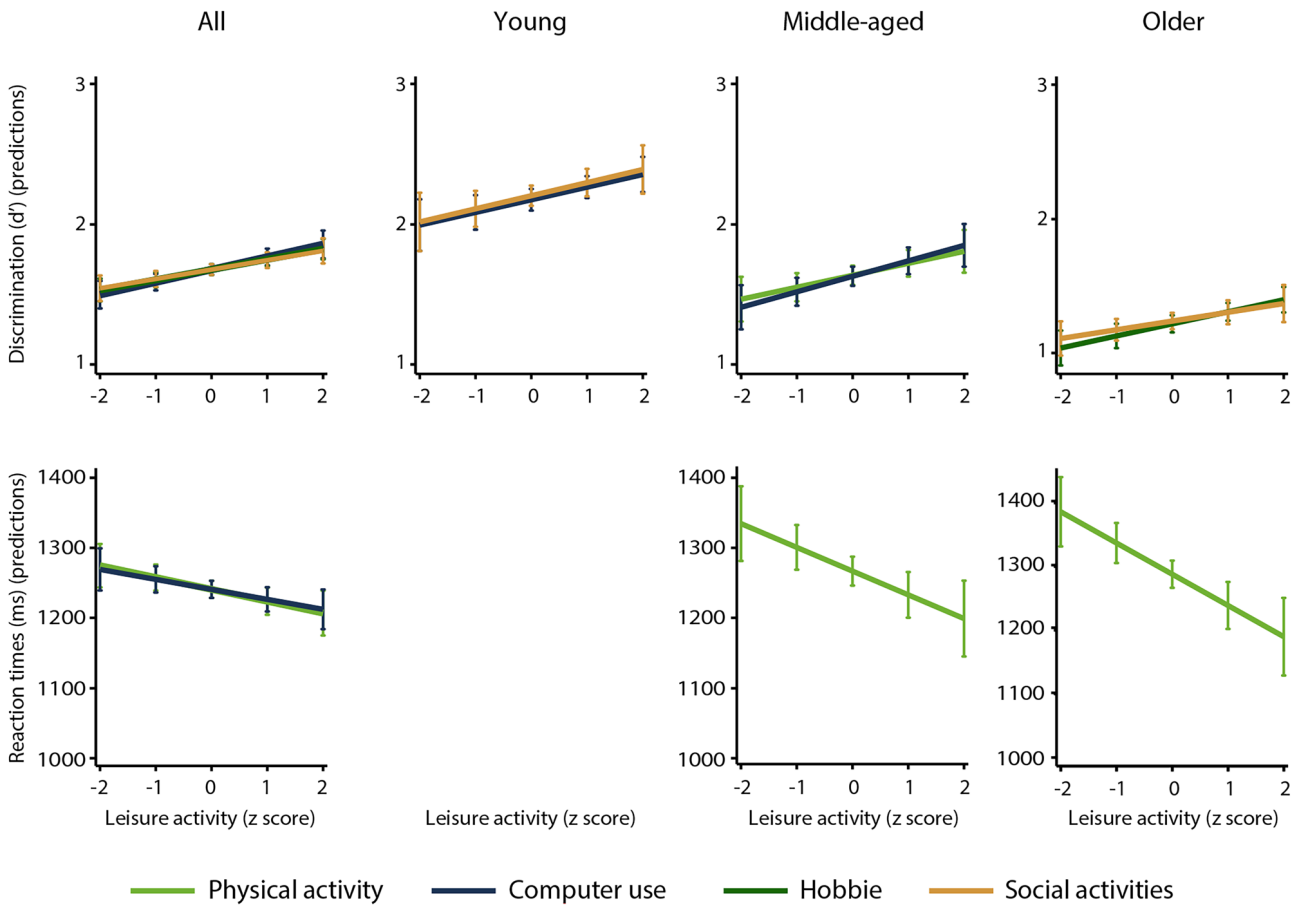
Regression plots for leisure activities that significantly predicted discrimination levels and reaction times for spatial working memory in the entire sample and in each age group. Leisure activities are depicted in z scores to allow for comparison. Error bars represent the 95% confidence intervals of the mean

Multiple regression models conducted on data from the entire sample revealed that mental (computer use and hobbies) and social leisure activities predicted greater verbal and spatial working memory discrimination. Faster verbal working memory responses were predicted by mental (reading) and social leisure activities, while faster spatial working memory responses were predicted by physical and mental (computer use) activities. These results are displayed in Table 2. The analyses performed on data from the young adults showed that mental (computer use) and social leisure activities were associated with greater verbal and spatial working memory discrimination, and social activity was also associated with greater verbal working memory speed (Table 3). The results of the analyses of data from middle-aged adults revealed that physical activity predicted greater spatial working memory discrimination and speed in verbal and spatial working memory, whereas mental activity (computer use) predicted greater verbal and spatial working memory discrimination and verbal working memory speed (Table 4). The results from the older adults showed that mental leisure activity (hobbies) was associated with greater verbal and spatial working memory discrimination, social activity was associated with verbal working memory, and physical activity was associated with visual and verbal working memory speed (Table 5). Figure 2 shows regression plots for leisure activities that significantly predicted discrimination levels and reaction times for verbal working memory in the entire sample and in each age group. Those that significantly predicted spatial working memory performance are displayed in Fig. 3. Leisure activities are presented in z scores to allow for an equivalent comparison.

**Table 2:** Multiple linear regression analyses predicting working memory discrimination and reaction time performance in the entire sample (n = 1652)

	Verbal						Spatial					
	Discrimination (d’)			Reaction time (ms)			Discrimination (d’)			Reaction time (ms)		
	*B*	*SE B*	*β*	*B*	*SE B*	*β*	*B*	*SE B*	*β*	*B*	*SE B*	*β*
Age (y)	− 0.02	0.00	− .41***	3.50	0.38	.26***	− 0.02	0.00	− .39***	3.03	0.44	.21***
Education (y)	0.01	0.01	.05	1.32	1.64	.02	0.03	0.01	.12***	0.12	1.80	.00
V (WAIS-R)	0.06	0.01	.11***	6.67	3.74	.05	0.06	0.01	.10***	9.00	4.08	.06*
MMSE	0.06	0.02	.08***	-2.78	4.41	− .02	0.04	0.01	.06**	1.22	4.95	.01
BDI	− 0.01	0.00	− .06**	0.31	1.19	.01	− 0.01	0.00	− .04	− 1.11	1.32	− .02
Physical A^a^	0.00	0.01	.01	− 3.21	2.51	− .03	0.01	0.01	.01	− 6.71	2.94	− .06*
Computer^a^	0.00	0.00	.07**	− 0.68	0.37	− .05	0.01	0.00	.10***	− 0.86	0.41	− .05*
Hobbies^a^	0.01	0.00	.06**	− 1.09	1.27	− .02	0.02	0.00	.08***	− 1.13	1.42	− .02
Reading^a^	0.00	0.00	− .01	− 1.74	0.72	− .06*	0.00	0.00	.01	− 1.11	0.81	− .04
Social A^b^	0.03	0.01	.06**	− 7.01	3.32	− .06*	0.04	0.01	.07***	− 3.20	3.58	− .02
Cultural A^b^	− 0.01	0.01	− .02	− 2.80	2.99	− .03	− 0.01	0.01	− .03	− 0.36	3.17	.00
Television^a^	0.00	0.00	.01	− 0.50	0.59	− .02	0.00	0.00	.03	− 0.17	0.59	− .01
Radio^a^	0.00	0.00	− .02	0.19	0.35	.01	0.00	0.00	− .01	0.40	0.37	.03
*R*^*2*^	.27			.10			.29			.06		
*F*	53.67***				13.54***		53.97***			8.38***		

*V* vocabulary subscale, *WAIS-R* Wechsler adult intelligence scale-revised, *MMSE* Mini mental state examination, *BDI* beck depression inventory, *A* activity

^a^Total time = frequency × duration

^b^Frequency

**Table 3:** Multiple linear regression analyses predicting working memory discrimination and reaction time performance in young adults (n = 527)

	Verbal						Spatial					
	Discrimination (d’)			Reaction time (ms)			Discrimination (d’)			Reaction time (ms)		
	*B*	*SE B*	*β*	*B*	*SE B*	*β*	*B*	*SE B*	*β*	*B*	*SE B*	*β*
Age (y)	− 0.04	0.01	− .32***	6.58	1.71	.18***	− 0.03	0.01	− .24***	7.39	1.91	.18***
Education (y)	0.02	0.01	.07	2.80	4.01	.03	0.03	0.02	.09	2.76	4.23	.03
V (WAIS-R)	0.04	0.02	.09	− 0.72	6.51	− .01	0.04	0.03	.07	0.71	7.29	.00
MMSE	0.06	0.03	.09*	− 5.06	9.41	− .03	0.05	0.03	.07	− 4.85	9.53	− .02
BDI	− 0.01	0.01	− .04	0.18	1.93	.00	0.00	0.01	.00	− 1.83	2.18	− .04
Physical A^a^	0.01	0.01	.03	2.56	3.76	.04	0.00	0.01	.01	0.12	4.39	.00
Computer^a^	0.00	0.00	.11*	− 0.22	0.54	− .02	0.01	0.00	.12**	− 0.78	0.59	− .06
Hobbies^a^	0.01	0.01	.06	− 4.58	2.45	− .08	0.01	0.01	.04	− 1.68	3.21	− .02
Reading^a^	0.00	0.00	− .04	− 2.06	1.18	− .08	0.00	0.00	.03	− 1.93	1.37	− .07
Social A^b^	0.05	0.02	.09*	− 17.13	5.90	− .12**	0.05	0.02	.09*	− 10.10	6.52	− .07
Cultural A^b^	− 0.01	0.02	− .02	− 7.17	6.18	− .06	− 0.02	0.02	− .03	− 4.26	6.15	− .03
Television^a^	0.00	0.00	.00	− 0.78	1.08	− .03	0.00	0.00	.04	0.10	1.20	.00
Radio^a^	0.00	0.00	− .04	− 0.20	0.52	− .02	0.00	0.00	.01	0.11	0.56	.01
*R*^*2*^	.17			.09			.12			.06		
*F*	7.60***				4.04***		5.41***			2.58**		

*V* vocabulary subscale, *WAIS-R* Wechsler adult intelligence scale-revised, *MMSE* mini mental state examination, *BDI* Beck depression inventory, *A* activity

^a^Total time = frequency × duration

^b^Frequency

**Table 4:** Multiple linear regression analyses predicting working memory discrimination and reaction time performance in middle-aged adults (n = 537)

	Verbal						Spatial					
	Discrimination (d’)			Reaction time (ms)			Discrimination (d’)			Reaction time (ms)		
	*B*	*SE B*	*β*	*B*	*SE B*	*β*	*B*	*SE B*	*β*	*B*	*SE B*	*β*
Age (y)	− 0.02	0.01	− .15***	2.11	1.90	.05	-0.01	0.01	− .09*	0.82	2.08	.02
Education (y)	0.03	0.01	.14**	− 1.88	2.58	− .04	0.03	0.01	.16**	− 3.06	2.84	− .06
V (WAIS-R)	0.05	0.02	.09*	19.10	6.64	.15**	0.07	0.02	.13**	10.59	7.22	.07
MMSE	0.05	0.03	.08	9.33	6.94	.06	0.04	0.03	.06	10.40	8.36	.06
BDI	− 0.01	0.01	− .08	1.47	2.11	.03	-0.01	0.01	− .05	0.78	2.28	.02
Physical A^a^	0.02	0.02	.05	− 11.19	5.01	− .11*	0.03	0.01	.09*	− 13.45	5.01	− .12**
Computer^a^	0.00	0.00	.09*	− 1.34	0.58	− .11*	0.01	0.00	.14***	− 1.08	0.63	− .08
Hobbies^a^	0.00	0.01	.01	1.72	2.64	.04	0.01	0.01	.05	− 0.38	2.98	− .01
Reading^a^	0.00	0.01	− .01	− 0.31	1.49	− .01	0.00	0.01	-.01	1.00	1.58	.03
Social A^b^	0.03	0.02	.06	− 1.16	5.97	− .01	0.01	0.02	.03	− 0.94	6.46	− .01
Cultural A^b^	− 0.03	0.02	− .06	− 2.52	5.45	− .02	− 0.01	0.02	− .03	1.40	5.78	.01
Television^a^	0.01	0.00	.08	− 0.77	0.95	− .03	0.00	0.00	.05	0.14	1.04	.01
Radio^a^	0.00	0.00	.01	0.80	0.52	.06	0.00	0.00	.02	0.40	0.60	.03
*R*^*2*^	.11			.05			.14			.03		
*F*	5.44***				2.31**		7.26***			1.17		

*V* vocabulary subscale, *WAIS-R* Wechsler adult intelligence Scale-Revised, *MMSE* mini mental state examination, *BDI* Beck depression inventory, *A* activity

^a^Total time = frequency × duration

^b^Frequency

**Table 5:** Multiple linear regression analyses predicting working memory discrimination and reaction time performance in older adults (n = 588)

	Verbal						Spatial					
	Discrimination (d’)			Reaction time (ms)			Discrimination (d’)			Reaction time (ms)		
	*B*	*SE B*	*β*	*B*	*SE B*	*β*	*B*	*SE B*	*β*	*B*	*SE B*	*β*
Age (y)	− 0.01	0.01	− .08*	− 0.87	1.91	− .02	− 0.02	0.01	− .16***	− 2.75	2.04	− .06
Education (y)	0.00	0.01	.02	1.48	2.67	.03	0.03	0.01	.16***	− 1.22	2.93	− .02
V (WAIS-R)	0.06	0.02	.13**	8.84	6.32	.07	0.05	0.02	.11**	20.42	6.94	.14**
MMSE	0.06	0.02	.11**	− 11.06	6.83	− .07	0.04	0.02	.07	− 5.79	7.96	− .03
BDI	− 0.01	0.01	− .09*	0.25	2.07	.01	− 0.02	0.01	− .10**	− 0.82	2.27	− .02
Physical A^a^	− 0.01	0.02	− .03	− 11.55	3.87	− .10**	− 0.02	0.02	− .05	− 19.49	5.43	− .15***
Computer^a^	0.01	0.01	.06	− 2.50	1.56	− .07	0.01	0.01	.05	− 2.94	1.75	− .07
Hobbies^a^	0.02	0.01	.11**	− 1.17	1.61	− .03	0.02	0.01	.13***	− 1.26	1.54	− .03
Reading^a^	0.00	0.00	− .03	− 0.99	1.31	− .03	0.00	0.00	− .03	0.33	1.48	.01
Social A^b^	0.01	0.01	.02	− 4.56	5.36	− .04	0.03	0.02	.09*	1.90	5.51	.02
Cultural A^b^	− 0.01	0.01	− .03	0.54	4.56	.01	-0.02	0.02	− .06	3.45	4.83	.03
Television^a^	0.00	0.00	− .03	− 0.43	1.00	− .02	0.00	0.00	.00	− 0.60	0.96	− .02
Radio^a^	0.00	0.00	− .02	− 0.55	0.74	− .04	0.00	0.00	− .06	0.08	0.75	.00
*R*^*2*^	.08			.02			.14			.04		
*F*	3.71***				1.44		8.83***			1.88*		

*V* vocabulary subscale, *WAIS-R* Wechsler adult intelligence scale-revised, *MMSE* mini mental state examination, *BDI* beck depression inventory, *A* activity

^a^Total time = frequency × duration

^b^Frequency

**Fig. 4:**
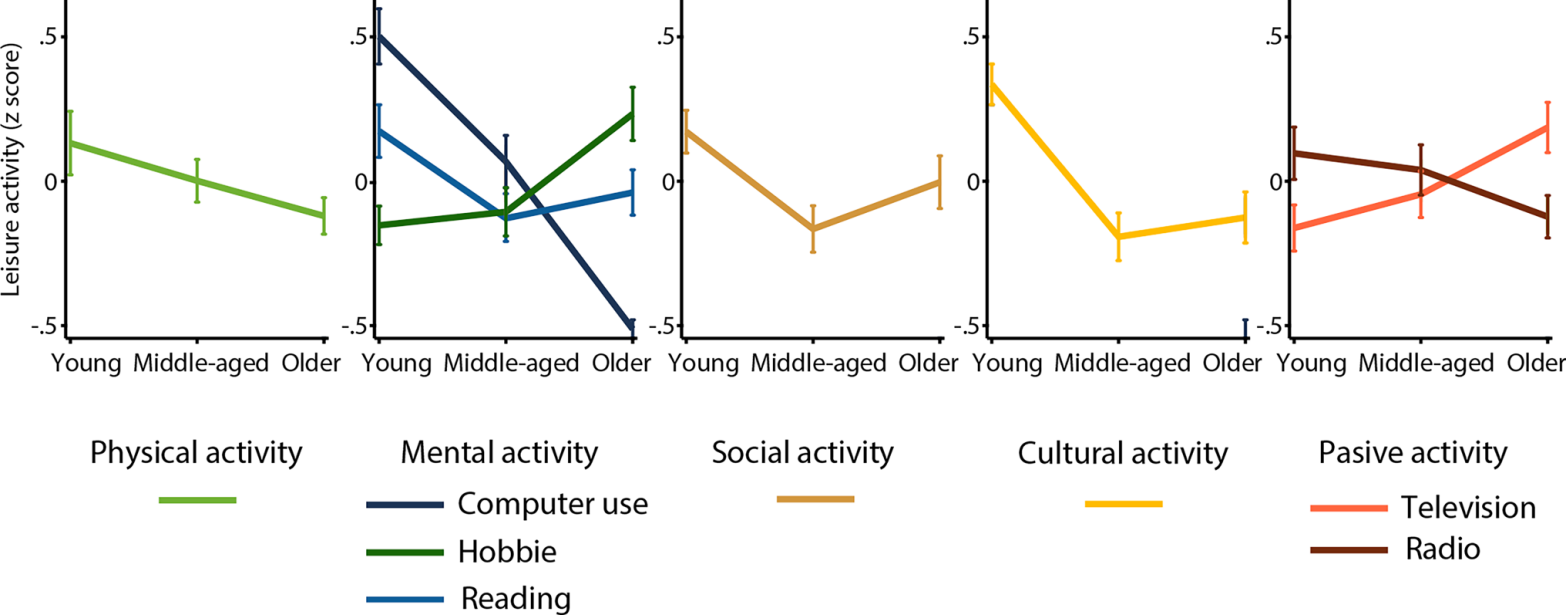
For each leisure activity, the mean activity is displayed for young, middle-aged and older adults. Leisure activities are displayed in z scores to allow for comparison. Error bars represent the 95% confidence intervals of the mean

Throughout the adult life span, engagement in all leisure activities changed. Engagement in each leisure activity for young, middle-aged, and older adults is displayed in z scores in Fig. 4. With regard to physical activity [*F*(2, 1649) = 8.93, *p* < 0.001, μ^2^ = 0.01], Tukey HSD tests revealed that young adults (mean ± standard error = 1.07 ± 0.11) dedicated more time to performing leisure physical activity than older adults (0.44 ± 0.10), but middle-aged adults’ (0.74 ± 0.11) engagement in physical activity did not differ from that of young and older adults. Concerning computer use [*F*(2, 1649) = 175.16, *p* < 0.001, μ^2^ = 0.18], post hoc analysis revealed that computer use decreased significantly in young (18.95 ± 0.66), middle-aged (11.74 ± 0.65) and older adults (2.11 ± 0.62). With respect to reading [*F*(2, 1649) = 13.01, *p* < 0.001, μ^2^ = 0.02], Tukey’s HSD test revealed that young adults (9.10 ± 0.37) read more than middle-aged (6.53 ± 0.36) and older adults (7.29 ± 0.35). According to post hoc analyses, engagement in hobbies [*F*(2, 1649) = 25.93, *p* < 0.001, μ^2^ 0.03] was less common in young adults (1.80 ± 0.21) than in older adults (3.65 ± 0.19), and middle-aged adults (2.02 ± 0.20) engaged in hobbies less often than older adults. Concerning social activities [*F*(2, 1649) = 15.38, *p* < 0.001, μ^2^ = 0.02], post hoc analyses showed that young adults (4.56 ± 0.08) attended more social events than middle-aged (3.93 ± 0.08) and older (4.23 ± 0.08) adults did, while older adults attended more social events than middle-aged adults. Regarding cultural activities [*F*(2, 1649) = 46.66, *p* < 0.001, μ^2^ = 0.05], post hoc tests revealed that young adults (4.43 ± 0.09) attended more cultural events than middle-aged (3.29 ± 0.09) and older (3.44 ± 0.09) adults. With regard to television watching [*F*(2, 1649) = 18.02, *p* < 0.001, μ^2^ = 0.02], post hoc analyses revealed that young adults (12.19 ± 0.43) watched less television than older adults (15.70 ± 0.41), and middle-aged adults (13.37 ± 0.43) watched less television than older adults. Regarding listening to the radio [*F*(2, 1649) = 7.37, *p* = *0.001*, μ^2^ = 0.01], post hoc tests revealed that young adults (19.33 ± 0.75) listened to the radio more than older adults (15.56 ± 0.71), and middle-aged adults (18.34 ± 0.74) listened more than older adults.

## Discussion

Specific leisure activities are associated with better verbal and spatial working memory discrimination and speed across the adult lifespan. Moreover, during each life stage of adulthood, different free-time activities were associated with working memory discrimination or speed. Additionally, the time spent on each leisure activity changed across adulthood. Below, we discuss each of these findings in detail.

Verbal and spatial working memory discrimination decrease significantly across young, middle-aged, and older adults; this phenomenon has been confirmed by several studies (e.g., Cansino et al., 2013; Park et al., 2002). Verbal working memory processing speed also decreased across all age groups, whereas spatial working memory processing speed decreased between young and middle-aged adults but not between middle-aged and older adults. This finding could be related to the high difficulty of the spatial task that induces more variable reaction times due to uncertainty, which would be more manifested as discrimination decreases.

### Physical activity

For middle-aged adults only, physical activity predicted greater spatial working memory discrimination. This occurred even when the total time spent in physical activity in the middle-aged group did not differ from that of the other two groups, although there was a clear tendency across adulthood toward decreasing physical activity. Thus, these findings were not related to the amount of time spent in physical activity but rather to the age group itself. Young adults probably did not benefit because they were already at their optimal working memory discrimination levels, and older adults’ working memory discrimination probably did not benefit from physical activity because the type of physical activity they performed did not reach the intensity required to have an effect on cognition. Only moderate-to-vigorous physical activity has shown positive effects on cognition (Engeroff et al., 2018; Willey et al., 2016). The older adults in the present study reported that the most common physical activity they performed was walking. Walking slowly is considered a low-intensity activity because the metabolic equivalent (MET) is approximately 2.0; to achieve moderate-intensity exercise, at least 5.0 METs are required, which would require walking one mile in 15 min (American College of Sports Medicine, 2006).

Therefore, in contrast to our prediction, working memory discrimination in older adults was not influenced by physical activity. This prediction was based on intervention studies (Zhidong et al., 2021) that controlled the exact amount of physical activity performed by older adults during a certain period of time. Conversely, we measured the actual physical activity that individuals choose to freely perform as a habit. This essential difference may explain the different outcomes. However, physical activity was associated with faster spatial working memory in the entire sample and with faster verbal and spatial working memory processing in middle-aged and older adults. Regular physical activity has been associated with faster simple and choice reaction times (for a review, see Spirduso, 1980). This review proposes that one of the mechanisms that may support faster responses is that physical activity preserves cardiovascular efficiency, which in turn increases blood flow and oxidative capacity in motor brain regions that increase metabolic capacity due to physical activity demands, thus improving the regions that support psychomotor speed. However, the current findings confirm that physical activity not only improves the speed of motor areas responsible for simple reaction times but also the speed of high-level cognitive processes, such as working memory. Therefore, the mechanisms underlying the positive effects of physical activity are not limited to motor areas. Physical activity enhances oxygen uptake, which is a prominent precursor of increased brain vasculature that benefits cognition and increases gray matter volume in brain regions including the prefrontal cortex (Miller et al., 2012), which is a crucial region for working memory.

### Mental activity

The time per week that individuals dedicated to engaging in mental leisure activities (computer use and hobbies) was a powerful predictor of verbal and visual working memory discrimination in the entire sample after controlling for age, years of education, and the scores obtained in the vocabulary subtest of the WAIS-R, MMSE, and BDI. The effects of computer use and hobbies were slightly greater for spatial working memory than for verbal working memory, as revealed by the probabilities in the models, but were quite similar within each domain. The finding that mental leisure activity benefits both verbal and spatial working memory indicates that the positive effects of mental leisure activities are not limited only to the separate storage and rehearsal of verbal and spatial information but also involve central executive functioning, which is responsible for control processes within the working memory system, such as assigning resources and allocating attention.

A positive association between computer use and verbal and spatial working memory discrimination was observed in young and middle-aged adults when its effects were examined by adulthood stage. Older adults showed no benefit from this leisure activity, which could be explained by the fact that older adults spent significantly less time using computers than middle-aged adults did and therefore spent less time than young adults. Although middle-aged adults used computers less than young adults did, they still spent enough time to benefit from this leisure activity. In fact, the association between computer use and spatial working memory was stronger in middle-aged adults than in young adults, indicating that with advancing age, the benefit of using a computer increases.

Older adults benefited from hobbies, whereas the other two age groups did not. The reason could be attributed to the time spent on hobbies, which was significantly less for young and middle-aged adults than older adults. This finding supports our prediction that older adults benefit from mental activities. In fact, performing hobbies is highly cognitively demanding because it involves effort and coordination among different systems, such as the cognitive, motor and sensory systems. Our findings are in accordance with other studies that found that engaging in hobbies has a positive effect on general cognition (Wang et al., 2013a, 2013b). Therefore, mental leisure activities influence working memory as predicted, but their effects are strongly related to the time spent on them, and each stage of adulthood is involved in a particular mental activity.

Reading was associated with verbal working memory speed in the entire sample, whereas computer use was associated with spatial working memory speed. Both outcomes were theoretically expected. Several processes are activated during reading; some are considered low level, such as word recognition and syntactic parsing, and some are considered high level, such as comprehension and inference. These processes rely on attention, working memory, executive control, and metacognition (Wylie et al., 2018). The relationship between working memory and reading has been extensively investigated in children. For example, a meta-analysis (Peng et al., 2018) of 197 studies revealed a significant relationship between reading and working memory. However, our findings go beyond these previous findings because we found a relationship between reading and the speed of processing verbal information within working memory, not with working memory accuracy, as has been frequently observed. Moreover, we found this relationship in adults but not in children. This outcome is theoretically expected because the more time people dedicate to reading, the more fluent reading becomes and the more automated the processes involved in reading become (Ashby & Rayner, 2006). Consequently, fewer working memory demands are needed, especially for processing verbal information faster, as was observed in the present study.

Numerous studies have shown that the use of computers to play video games for entertainment, educational, or cognitive challenge purposes enhances spatial abilities (for a review, see Barlett et al., 2009). Older adults trained with computerized video games showed improvements in several cognitive functions, as revealed by two meta-analyses. One meta-analysis included 20 studies (Toril et al., 2014) and reported positive effects on memory (all types of memories were analyzed jointly) and simple reaction time. The other meta-analysis examined 16 studies (Bonnechère et al., 2020) and revealed positive effects on working memory and processing speed. The positive effects of computer use have been observed in intervention studies, mainly in older adults. Therefore, the current findings contribute to knowledge of this leisure activity because they confirm that the use of computers in real life for all purposes, including playing video games, enhances spatial working memory speed across adulthood and does not benefit only one life stage.

### Social activities

Social leisure activities predicted greater verbal and spatial working memory discrimination in the entire sample. This outcome confirms our predictions and is in agreement with the findings of a meta-analysis (Kelly et al., 2017) that reported an association between social activity and working memory in adults older than 50. When analyzing each adulthood stage, social activity was also associated with greater verbal and spatial working memory discrimination in young adults. This finding has not been previously reported because the effects of leisure activities on cognitive function in young adults have not been examined previously. Young adults reported that they engaged in more social activities than middle-aged and older adults, and older adults engaged in more social activities than middle-aged adults. In fact, older adults’ social activity was also associated with greater working memory but only in the spatial domain. Therefore, the results indicate that social activity is a powerful predictor of working memory at any age, but its influence depends on how often individuals attend social events. Although social activity showed effects on the entire sample, middle-aged adults showed no working memory benefit associated with social activity because this age group practically omitted this leisure activity from their lives. Older adults’ positive association between social activity and working memory was limited to the spatial domain because their social activity was not frequent enough to benefit both domains.

Social leisure activity has been associated with brain volume changes. In a randomized trial (Mortimer et al., 2012) in which healthy older adults engaged in social reunions for one hour three times a week for 40 weeks, white and gray matter volumes increased compared to a no-intervention group. In the present study, we also observed that attending social events was associated with verbal working memory speed in the entire sample and in young adults. A meta-analysis (Brown et al., 2012) that examined four longitudinal studies revealed that social activity was associated with greater verbal fluency in older adults. This study concluded that verbal fluency improved because social activity requires verbal communication abilities. This finding is in agreement with the current results because rapidly processing verbal information within working memory also requires verbal fluency, which is enhanced by engaging in social events.

### Cultural activities

Cultural leisure activities were not associated with either working memory discrimination or speed in the present study. Although young adults attended cultural events more often than middle-aged and older adults did, this leisure activity was not associated with working memory in this age group. The lack of association between attending cultural events and working memory contradicts previous findings (Fancourt & Steptoe, 2018) in which attending cultural events was associated with higher general measures of memory. The assessment of this leisure activity should be more specific to determine the lack of agreement between studies because attending different cultural activities, such as film screenings, theater plays, exhibitions, concerts, conferences, or courses, might not have the same impact or might not be selected with the same probability across adulthood.

### Passive activities

As expected, watching TV and listening to the radio did not influence working memory discrimination or speed. The fact that these activities had no effect on working memory confirms the passive nature of these activities and contrasts with their classification as mental effortful activities (Hassing et al., 2020). Moreover, the present results contribute to the controversy due to the opposite results observed with this type of recreational activity (Mao et al., 2109; Raichlen et al., 2022) because we found neutral effects. TV watching did not affect working memory in a previous study (O’Connor et al., 2016) in which the time children between 6 and 9 years spent watching TV was not associated with working memory performance at the age of 14 years after controlling for several social characteristics. The present findings confirm that engaging in leisure activities that do not represent a cognitive challenge has no effect on highly demanding cognitive functions, such as working memory. This information is important to consider when individuals choose how to spend their free time. Moreover, time spent watching TV increases across young, middle-aged, and older adults, which implies that older adults spend more time performing a leisure activity that has no benefit for working memory, one of the most crucial types of memory for dealing with everyday living challenges. Time spent listening to the radio showed the opposite trend and decreased across young, middle-aged, and older adults. However, because young adults’ working memory is at its highest level, as revealed by studies that compare working memory performance across adult lifespans (Cansino et al., 2013; Park et al., 2002), spending more time in a passive activity has no consequences for this group.

### Limitations

One limitation of the study is that the sample size was not calculated a priori and was determined by financial resource constraints. However, the fact that we found significant results confirmed that the sample size had sufficient power to detect effects. As an observational study, another limitation of the present research is that leisure activities were assessed through the participants’ reports, which may be affected by inaccuracies, inexact memory, and social desirability. This type of measurement error corresponds to the dimension of accuracy—in particular, validity—which is a relevant dimension to consider when performing quality surveys (Di Leonardo et al., 2017) but may also describe the typical errors encountered when performing observational studies in which information depends on participants’ reports. However, these random measurement errors were distributed among all participants. As in any cross-sectional study, the causal influence of leisure activities on working memory cannot be confirmed. Although leisure activities were present before memory was tested, the direction of causality between variables remains to be confirmed through longitudinal studies. Despite these limitations, observational studies have the advantage of studying variables as they actually occur in real life. The data obtained in the source memory task will be a future line of work that will improve our knowledge of the influence of leisure activity on episodic memory, which has been scarcely investigated (Yang et al., 2022).

## Conclusions

We were able to identify the specific leisure activities that are associated with better verbal and spatial working memory discrimination and speed in a lifespan sample and in specific stages of adulthood. Mental and social leisure activities were the most influential predictors of working memory discrimination across the entire lifespan and for all age groups. Although the specific mental activity that benefits each age group is different, computer use benefited young and middle-aged adults, whereas engaging in hobbies benefited older adults. These leisure activities were more important for specific age groups because these were the activities in which they were more involved; however, the potential influence of leisure activities on working memory discrimination extends through the entire life span. Time spent reading was associated with the speed of processing verbal information within working memory, whereas computer use was associated with the speed of processing spatial information. Physical activity was mostly associated with working memory speed in both domains, especially in middle-aged and older adults. The present findings reveal the powerful effect of leisure-time activities on working memory.

## Supplementary Information

Below is the link to the electronic supplementary material.

Supplementary file1 (PDF 34 KB)

## Data Availability

The data are available upon reasonable request.
