# Supplementary material for: Effects of different types of leisure activities on working memory across the adult lifespan
Source: Psychol Res. 2024 Jul 6;88(7):1981–95. doi: 10.1007/s00426-024-01998-x (PMC11449988; doi:10.1007/s00426-024-01998-x)
Supplement: Supplementary file 1 — Supplementary file1 (PDF 34 KB) [file 426_2024_1998_MOESM1_ESM.pdf]

SUPPLEMENTARY INFORMATION

Effects of different types of leisure activities on working memory across the adult lifespan

Selene Cansino*, Frine Torres-Trejo, Cinthya Estrada-Manilla, Frine Torres-Trejo and Silvia Ruiz-Velasco

Supplementary Table S1. Descriptive statistics.

	Mean or number	*SD*	Skewness	Kurtosis
Age (years)	51.02	17.71	-0.13	1.80
Sex (women/men)[a]	831/821			
Education (years)	14.52	4.23	0.48	3.34
Vocabulary scale (WAIS-R)	12.71	1.71	0.11	2.70
Mini Mental State Examination	28.54	1.38	-0.89	3.29
Beck Depression Inventory	6.61	5.08	0.68	2.62
Physical activity[b]	0.74	2.52	4.92	31.81
Television[b]	13.82	10.07	1.79	8.81
Radio[b]	17.66	17.19	1.58	5.59
Computer use[b]	10.61	16.62	1.80	5.77
Reading[b]	7.62	8.50	2.34	10.92
Hobbies[b]	2.53	4.78	3.12	17.15
Cultural activities (fr)	3.71	2.16	-0.44	2.14
Social activities (fr)	4.24	1.88	-0.55	2.94
Verbal working memory (d')	2.14	0.91	-0.04	2.54
Spatial working memory (d')	1.68	0.93	0.08	2.44
Verbal working memory RT (ms)	1137	239	0.14	2.99
Spatial working memory RT (ms)	1240	260	-0.03	2.85

Note. [a] Dichotomous variable

[b] Total time = frequency x duration

fr = frequency, WAIS-R = Wechsler Adult Intelligence Scale-Revised

Supplementary Table S2. Correlations among variables in the total sample.

	1	2	3	4	5	6	7	8	9	10	11	12	13	14	15	16	17
1. Age (years)	—																
2. Education (years)	-.27***	—															
3. V. (WAIS-R)	-.02	.37***	—														
4. MMSE	-.30***	.16***	.19***	—													
5. BDI	.09***	-.23***	-.16***	-.05*	—												
6. Physical activity[a]	-.09***	.11***	.03	.01	-.06*	—											
7. Television[a]	.15***	-.20***	-.09***	-.06*	.11***	-.05	—										
8. Radio[a]	-.07**	-.02	-.06*	.01	.05*	-.03	.03	—									
9. Computer use[a]	-.39***	.32***	.11***	.15***	-.16***	.06*	-.17***	.06**	—								
10. Reading[a]	-.09***	.35***	.22***	.08***	-.17***	.06*	-.16***	.01	.21***	—							
11. Hobbies[a]	.17***	-.10***	.00	-.05*	.00	-.02	.08**	.03	-.11***	.02	—						
12. Cultural activities[b]	-.21***	.36***	.21***	.15***	-.17***	.08**	-.07**	.06*	.18***	.23***	.05*	—					
13. Social activities[b]	-.08***	.09***	.05*	.02	-.10***	.05	.00	.03	.04	.02	.03	.25***	—				
14. VWM (d’)	-.47***	.24***	.17***	.24***	-.15***	.07**	-.08***	.01	.27***	.10***	-.02	.16***	.11***	—			
15. SWM (d’)	-.47***	.30***	.18***	.22***	-.14***	.07**	-.08**	.02	.30***	.13***	-.01	.17***	.13***	.55***	—		
16. VWM RT (ms)	.29***	-.08***	.02	-.09***	.05	-.06**	.03	-.02	-.16***	-.09***	.02	-.10***	-.08***	-.33***	-.23***	—	
17. SWM RT (ms)	.23***	-.06**	.04	-.05*	.01	-.09***	.03	.00	-.13	-.05*	.02	-.06*	-.04	-.17***	-.27***	.60***	—

Note. [a] Total time = frequency x duration
[b] Frequency
V. = Vocabulary subscale, WAIS-R = Wechsler Adult Intelligence Scale-Revised, MMSE = Mini Mental State Examination, BDI = Beck Depression Inventory, VWM = verbal working memory, SWM = spatial working memory, RT = reaction time
* $p < 0.05$, ** $p < 0.01$, *** $p < 0.001$
